# Associations between Blood Zinc Concentrations and Sleep Quality in Childhood: A Cohort Study

**DOI:** 10.3390/nu7075247

**Published:** 2015-07-13

**Authors:** Xiaopeng Ji, Jianghong Liu

**Affiliations:** School of Nursing University of Pennsylvania, 418 Curie Blvd., Philadelphia, PA 19104, USA; E-Mail: jixiaop@nursing.upenn.edu

**Keywords:** blood zinc concentration, sleep quality, preschool children, adolescents, micronutrients

## Abstract

Little evidence is available regarding the relationship between zinc and sleep in school children. The present study aimed to examine the cross-sectional and longitudinal associations between blood zinc concentrations and sleep quality throughout childhood. A total of 1295 children from the Jintan Child Cohort in China were included in this study. Venous blood sample of zinc and subjective sleep data were collected when the children were at preschool age (3–5 years old) and early adolescence (11–15 years old). Odds ratios (ORs) reflect the odds of the sleep quality/subdomain being at a greater impairment level associated with 1 unit increase in log zinc concentration. Cross-sectional analyses showed negative correlation of blood zinc concentrations with insufficient sleep duration (OR = 0.432, *p* = 0.002), sleep disturbances (OR = 0.454, *p* = 0.009) and poor sleep quality (OR = 0.559, *p* = 0.049) in adolescence, but no association at preschool age (*p* > 0.05). Longitudinal analyses indicated that blood zinc concentrations at preschool age predict poor sleep efficiency (OR = 0.186, *p* = 0.000) and poor sleep quality (OR = 0.358, *p* = 0.020) in adolescence. Our findings suggest that sufficient zinc concentration is associated with good sleep quality, dependent on the developmental stage in childhood. Future interventional research is warranted to examine the short and long-term effect of zinc status on sleep heath.

## 1. Introduction

Nutrient status in humans has received significant attention as a potentially modifiable factor of sleep quality over the past few decades [1,2]. For macronutrients, low proportion of carbohydrate intake was found to increase the percentage of slow wave sleep (deep sleep) and reduce the percentage of rapid eye movement (REM) sleep among healthy good sleepers [3]. In terms of micronutrients, the involvement of iron on sleep regulation is being increasingly investigated due to its possible role in regulating neurotransmitters essential to the intrinsic sleep processes [4,5]. Researchers generally supported a beneficial effect of iron supplement and normal blood iron concentrations on development of sleep architecture and longer sleep duration among infants [1,6,7]. The study by Grandner, Jackson, Gerstner, and Knutson extended the generalizability of the results to a sample in the general adult population, showing that decreased iron intake was associated with very short sleep (<5 h) after adjustment for overall diet [8].

Although zinc has not received as much attention as iron with respect to sleep outcomes, evidence has emerged that suggests a relationship of dietary zinc and blood zinc status with sleep quality and quantity. For example, the cross-sectional study by Grandner *et al.* has shown that decreased zinc intake aligns with very short sleep on a general adult population [8]. Similar links between zinc and sleep were also found in a study among infants [1]. However, since most of the studies used the level of dietary/supplement intake as a proxy for zinc status, biological zinc measures are thus far lacking in this field. Furthermore, most of the studies primarily focused on the short-term effect of zinc status on sleep patterns, or the cross-sectional correlations. The long-lasting effect of abnormal zinc status on sleep quality remains uncertain. 

Additionally, little evidence is available with respect to the relationship between zinc status and sleep quality in adolescents. Adolescence is often accompanied by developmental changes in sleep patterns, including a marked tendency for later bedtimes, insufficient sleep, long sleep-onset latency, and large night-to-night variability in sleep schedules [9,10,11]. Sleep problems during adolescence are prevalent globally, with between 14% and 68.9% of adolescents reporting disturbed sleep-wake function or impaired sleep quality [9,10,11,12]. Given the rapid brain growth and susceptibility to sleep impairment throughout adolescence [13], suboptimal zinc status could particularly impair intrinsic sleep regulation and sleep quality, and in turn affect adolescent health in general.

The aims of the present study were: to determine whether the concurrent association between blood zinc status and sleep quality was present at preschool age and adolescence respectively; and to examine whether blood zinc status at preschool age was predictive of sleep quality at adolescence. Understanding the cross-sectional and longitudinal association of zinc status with sleep quality will provide clues to understand the biological driven of sleep impairment in adolescents, and inform future interventions for the multifaceted and interrelated health issues of nutrition and sleep.

## 2. Materials and Methods

### 2.1. Participants and Procedures

This was a 9-year ancillary study nested within the China Jintan child cohort study. The city of Jintan is located in Jiangsu province, the southern region of China. When the parent study began in the fall of 2004, 1656 Chinese children (55.5% boys, 44.5% girls) aged 3–5 years old were recruited from four preschools in the city of Jintan [14]. Both blood zinc and sleep data collection took place in two waves at 2004–2005 and 2011–2013. We refer to the year 2004 to 2005 as “preschool age” and year 2011–2013 as “adolescence” throughout this paper. Detailed sampling and research procedures of this larger cohort study have been described elsewhere [14,15].

The present study used a subsample of children with both blood zinc concentrations and completed sleep data to test the cross-sectional and longitudinal relationships. Of 1656 participants in the initial cohort, 1295 children (3–5 years old) have both blood zinc concentration and sleep data available at preschool age. Some children dropped out in the follow-ups because they changed schools or because data were not fully available. The final data set used to address the concurrent association in adolescence was comprised of 777 children with a mean age of 13.16 years old (SD = 0.90, range =11–15). For the samples used to address the longitudinal association included 781 children whose blood zinc concentrations (preschool) and sleep data (preschool and adolescence) were available. Whereas grade did not differ between children with and those without data at adolescence (*χ*^2^ = 4.19, *p* > 0.05), differences were shown in sex as well as educational level in mother and father (*p* = 0.005–0.018). These variables were adjusted for in data analyses. Written informed consent was obtained from parents. Institutional Review Board (IRB) approval was obtained from the University of Pennsylvania and the ethical committee for research at Jintan Hospital in China.

### 2.2. Measures

#### 2.2.1. Blood Zinc Concentration

Blood specimens were collected by trained pediatric nurses using a strict research protocol at two time points: the first was in fall 2004-spring 2005 (preschool) when children were in preschool (3–5 years old); and the second was in summer 2011 to summer 2013 (adolescence) when they were in the last few months of 6th grade (11–15 years old). Approximately 0.5 mL of venous blood samples were collected in a lead-free EDTA tube. At preschool point, blood samples were frozen and shipped to the Child Development Center, Nanjing Medical University, Nanjing, China. Specimens were remained frozen at −20 °C before analyses were finalized. Zinc concentrations were determined by atomic absorption spectrophotometry (BH model 5.100 manufactured by Beijing Bohu Innovative Electronic Technology Corporation, Beijing, China), and duplicate readings were taken with an integration time of 2 s [16]. The reliability and validity of the procedure have been described previously [17]. At adolescence, a similar but updated analytical method was used in Xin Hua Hospital Affiliated to Shanghai Jiao Tong University School of Medicine, Shanghai, China. Specimens were restored at −40 °C until analysis using inductively coupled plasma mass spectrometry. The detailed analytical procedure was reported elsewhere [18].

#### 2.2.2. Sleep Measures

At preschool age, sleep quality of children was measured with sleep items in the Chinese version of Child behavior check list (CBCL) from the Achenbach System of Empirically Based Assessment (ASEBA) by parental report [19,20]. Parents answered questions about their child’s experiences within the past 12 months, and give a rating from a 3-point scale (0 = not true; 1 = sometimes true, or 2 = often true) [20]. Seven items on the CBCL specific to sleep were the basis for analyses in the present study, including unwilling to sleep alone, difficulty initiating sleep, having nightmares, resisting going to bed at night, sleeping less than most children, talking or crying out in sleep and difficulty maintaining sleep (DMS) [19]. The CBCL has been used to assess sleep problems in Chinese children and demonstrated satisfactory psychometric properties [21].

At adolescence, children were asked to fill out the Chinese version of the Pittsburgh Sleep Quality Index (CPSQI) in June–July 2013. CPSQI measures self-described sleep pattern and quality over the prior month. It is composed of 19 items that are scored to determine seven component scores: subjective sleep quality, sleep latency, sleep duration, sleep efficiency, sleep disturbances, use of sleeping medication and daytime dysfunction [22]. Scoring of sub-domains ranges from 0 to 3, and the PSQI total score from 0 to 21, with higher scores reflecting greater impairment [23]. Participants with total PSQI score greater than 5 is classified as poor sleep quality [23]. To make the PSQI more sensitive to adolescent sleep patterns, the present study recoded the domain scores of sleep duration as: 0 = longer than 9 h, 1 = 8–9 h, 2 = 7–8 h, and 3 = less than 7 h based on the cutoffs from the National Sleep Foundation (NSF). The PSQI was reported 0.87 for the overall reliability and ranged from 0.46 to 0.85 for subscales, and the cumulative variance of principal components was 70.72% in Chinese adolescents [22].

#### 2.2.3. Socio-Demographic Variables

The socio-demographic variables collected during each wave included sex, age, educational level in father and mother, as well as school. At adolescence, we collected grade of adolescents as well given its potential direct effect on sleep quality.

### 2.3. Statistical Analysis

Descriptive statistics, including frequencies and percentages, were used to characterize categorical demographic factors. Blood zinc concentrations were summarized by using means and standard deviations. Student *t* test and ANOVA were used to examine the association of blood zinc concentration with sample characteristics at the bivariate level. If there was significant difference among groups in ANOVA, the post hoc *Tukey’s* HSD test was used for multiple comparison. To address the association between zinc and sleep, we modeled blood zinc status as a continuous variable (log transformed concentration), because there was evidence of a linear association in previous research [24].

For the cross-sectional analysis, whereas the generalized linear model was used to examine the association between blood zinc concentrations and CBCL sleep scores at preschool, logistic regression models and ordinal logistic regression models were used to test the relationship of blood zinc concentration with concurrent poor sleep quality (PSQI total > 5) and seven sleep subdomains at adolescence. Models were adjusted for grade, sex, education level in mother and in father. Age was not included as a covariance due to the collinearity with grade. In terms of the longitudinal relationship, logistic regression models and ordinal logistic regression models were used to test the predictive effect of preschool zinc concentration on adolescent sleep outcomes, controlling for preschool sleep quality, sex, grade as well as education level in mother and father. Analyses were clustered by school. Statistical significance was taken at the two-sided *p* < 0.05 level. All the analyses were performed using STATA version 13.1.

## 3. Results

### 3.1. Sample Characteristics and Zinc Profile

The sample characteristic, sleep status and blood zinc profile at preschool were presented elsewhere by our research team [19,25]. Between 2011 and 2013, participants were in their early adolescence aged from 11 to 15 years old. Table 1 presents descriptive characteristics of socio-demographic variables and associated blood zinc concentrations at adolescence. Only grade had significant trends across zinc status, with blood zinc concentrations in grade 8 greater than that those in grade 7 (*p* = 0.006). According to the PSQI total score, 306 (39.38%) adolescents were classified as poor sleepers. Mean zinc concentrations at preschool and adolescence were described in Table 2 according to adolescent sleep status. Blood zinc concentrations at adolescence (87.92 ± 15.82 μg/dL) were significantly greater than those at preschool (82.26 ± 13.24 μg/dL) (*t* = −7.58, *p* = 0.000).

**Table 1:** Socio-demographic characteristics at adolescence.

	N(%)	Mean ± SD (μg/dL)	*t*/*F*	*p* value
Sex				
Boys	403(51.87)	88.63 ± 0.78	0.04	0.850
Girls	374(48.13)	82.96 ± 0.83		
Grade				
6	243(31.27)	87.78 ± 12.40	3.75	0.024 *
7	222(28.57)	90.20 ± 16.01		
8	312(40.15)	86.41 ± 17.79		
Mother’s education ^a^				
1. Middle school or less	318(42.57)	88.73 ± 15.55	0.78	0.457
2. High school	165(22.09)	86.84 ± 15.15		
3. College or higher	264(35.34)	87.86 ± 16.78		
Father’s education ^b^				
1. Middle school or less	222(29.68)	89.48 ± 14.91	1.56	0.210
2. High school	215(28.74)	86.91 ± 14.39		
3. College or higher	311(41.58)	87.63 ± 17.47		

^a^ variables have missing data, *n* = 747; ^b^. *N* = 748; * Turkey’s HSD test: blood zinc concentration in grade 8 > 7, *p* = 0.006.

**Table 2:** Preschool ^a^ and adolescent ^b^ blood zinc concentrations ^c^ by adolescent sleep quality.

Sleep Quality (Adolescence)	Zinc (Preschool)		Zinc (Adolescence)	
	*N*	Mean ± SD	*N*	Mean ± SD
Poor	314	81.15 ± 13.27	306	87.04 ± 15.90
Normal	467	83.17 ± 13.17	471	88.49 ± 15.75
Total	781	82.26 ± 13.24	777	87.92 ±15.82

^a^ Preschool: 3–5 years old in our sample; ^b^ Adolescent: 11–15 years old in our sample; ^c^ Unit of zinc concentration: μg/dL

### 3.2. The Cross-Sectional Association at Preschool and Adolescence

At preschool, the general linear model showed no cross-sectional association of blood zinc concentration with concurrent total score of CBCL sleep items at 3–5 years old (*p* > 0.05), after adjustment for sex as well as the education level of mother and father when children were at preschool age. Analyses were repeated with each CBCL sleep item by ordinal logistic regression model; all produced insignificant association (*p* > 0.05).

At adolescence, cross-sectional analyses found different statistical patterns. Table 3 shows the concurrent association of blood zinc concentrations with sleep outcomes in adolescents. Odds ratios (ORs) reflect the odds of the sleep quality/subdomain being at a greater impairment level associated with 1 unit increase in log transformed zinc concentration. Blood zinc concentrations at adolescence showed a negative association with the likelihood of concurrent poor sleep quality (OR = 0.559, *p* = 0.049), insufficient sleep duration (OR = 0.432, *p* = 0.002) and sleep disturbance (OR = 0.454, *p* = 0.009), independent of grade, sex, as well as education level in mother and father.

**Table 3:** Adjusted regression models ^a^ of sleep quality in adolescence on concurrent blood zinc concentrations ^b^.

Sleep Variables	OR (Robust SE)	95% CI	*P* values
Sleep subdomain ^d^			
Sleep duration	0.432 (0.115)	(0.257, 0.726)	0.002 **
Sleep disturbances	0.454 (0.138)	(0.250, 0.823)	0.009 **
Sleep latency	0.727 (0.139)	(0.499, 1.060)	0.097
Day dysfunction due to sleepiness	0.853 (0.287)	(0.441, 1.650)	0.637
Sleep efficiency	0.759 (0.361)	(0.299, 1.927)	0.563
Subjective sleep quality	0.705 (0.225)	(0.377, 1.319)	0.274
Sleep medication use	0.623 (0.472)	(0.141, 2.744)	0.532
Poor sleep quality ^c^	0.559 (0.164)	(0.314, 0.997)	0.049 *

^a^ Logistic regression model was used for poor sleep quality, and ordinal logistic regress models was used for sleep subdomains. Models controlled for sex, grade, education level of mother and education level of father, and clustered for schools; ^b^ Blood zinc concentrations were log transformed in the regression models; ^c^ Levels of sleep subdomains (except sleep duration): 0 = better, 3 = worse; a higher score indicates worse sleep quality; ^d^ Cutoff of poor sleep quality: total PSQI score > 5; ** p<0.01; *p<0.05

### 3.3. The Longitudinal Association

The blood zinc status at preschool was predictive of sleep quality in adolescence while adjusting for preschool sleep quality and socio-demographic variables. Specifically, the blood zinc concentrations at preschool age were negatively associated with the likelihood of poor sleep quality (overall) (OR = 0.349, *p* = 0.009) and poor sleep efficiency (OR = 0.186, *p* = 0.000) at adolescence. Table 4 presents the results from the longitudinal analyses.

**Table 4:** Adjusted regression models ^a^ of sleep quality in adolescence on blood zinc concentrations in preschool ^b^.

Sleep Variables	OR (Robust SE)	95% CI	*p* values
Sleep subdomain ^c^			
Sleep duration	1.187 (0.479)	(0.538, 2.621)	0.670
Sleep disturbances	1.103 (0.407)	(0.535, 2.275)	0.790
Sleep latency	0.870 (0.374)	(0.373,2.019)	0.742
Day dysfunction due to sleepiness	0.602 (0.185)	(0.330, 1.101)	0.100
Sleep efficiency	0.186 (0.088)	(0.073, 0.470)	0.000 **
Subjective sleep quality	0.560 (0.327)	(0.179, 1.756)	0.321
Sleep medication use	1.504 (1.718)	(0.160, 14.116)	0.721
Poor sleep quality ^d^	0.358 (0.159)	(0.150, 0.853)	0.020*

^a^ Logistic regression model was used for poor sleep quality, and ordinal logistic regress models was used for sleep subdomains. Models controlled for gender, grade, education level of mother and education level of father, preschool sleep quality, and clustered for schools; ^b^ Blood zinc concentrations were log transformed in the regression models; ^c^ Levels of sleep subdomains (except sleep duration): 0 = better, 3 = worse; a higher score indicates worse sleep quality; ^d^ Cutoff of poor sleep quality: total PSQI score > 5.

## 4. Discussion

The present study describes the cross-sectional and longitudinal association between zinc status and sleep outcomes in a normal school sample. Using data measured at preschool age (3–5 years old) and early adolescence (11–15 years old), our results indicated a number of significant associations between zinc status and childhood sleep, only some of which have been examined in previous studies. The cross-sectional analysis suggests a link between zinc concentrations and sleep quality in adolescence. Generally, adolescents with higher blood zinc concentrations were more likely to have normal sleep quality defined by PSQI total score. In terms of specific sleep domains, the likelihood of insufficient sleep and sleep disturbance decreased when zinc concentrations increased in adolescents. However, blood zinc status and sleep outcomes at preschool age showed no concurrent association. For the longitudinal analysis, lower blood zinc concentrations at preschool age predicted an increase in likelihood of poor sleep quality and worse sleep efficiency (ratio of sleep length/bed time) assessed in adolescence.

Very few studies have examined the overall sleep quality in relation to zinc status in humans, particularly in adolescents. In our sample, the cross-sectional findings at adolescence suggested that suboptimal blood zinc concentration may affect sleep duration and sleep disturbances, and in turn, impair the overall sleep quality among adolescents. Our findings of the relationship between zinc and sleep duration in adolescence are consistent with previous findings on other populations. For example, using maternal reports of sleep patterns, randomized controlled trials by Kordas *et al.* (2009) found longer night and total-sleep duration in infants receiving supplemental zinc relative to those in placebo group. However, whereas our study found a negative association between blood zinc concentration and the likelihood of insufficient sleep duration, the study on a sample of adult women with biomarkers of zinc status showed different trends [26]. This study observed the longest sleep hours in the middle tertile of serum zinc, and no association between hair zinc level and sleep duration among women [26]. Since this study defined the zinc level according to the distribution in 126 adult women recruited, the results over an arbitrary cutoff are prone to bias. In support to the associated sleep disturbances observed in our study, the benefit of dietary supplement of zinc, magnesium and melatonin has been tested as a treatment for primary insomnia in older adults, although the main effect of zinc alone was not reported [2].

The cross-sectional findings at adolescence are biologically plausible according to the discovery of potential biologic mechanisms from animal models. Zinc is a coenzyme required for neurogenesis, neuronal migration, and synaptogenesis [27]. Whereas zinc has been documented as an antagonist of excitatory transmissions, such as the *N*-methyl-d-aspartate receptor [28], zinc can also potentiate inhibitory transmissions of gamma-Aminobutyric acid (GABAA) receptors [29]. Given that neurotransmitters mentioned also relate to intrinsic sleep processes, zinc may moderate sleep quantity and quality through neurobiological pathways. Specific to adolescent animal models, zinc-deprived adolescent monkeys failed to show the shift to later initiation of the rest phase in late adolescence seen in the control group with normal diet [30]. Since delayed phase shift is a developmental marker of the sleep pattern during adolescence in humans [31] (pp. 142–144), zinc status can be a biological moderator of the developmental sleep change in adolescence.

In contrast to the significant concurrence in adolescence, a cross-sectional relationship between zinc and sleep was not present at preschool age, suggesting a developmental change in the correlation over time. This finding is consistent with studies on zinc and circadian rhythm, which showed significant links in adult women [24] but non-significant concurrence in children aged 6–8 years [32]. While micronutrient requirements [33] and normative sleep values [34] change across the lifespan, the relative predictive strength of zinc status may vary across early childhood, adolescence and adulthood. The present study observed this pattern in the same sample of children followed prospectively for 9 years, supporting that the concurrent association is moderated by development.

In addition to the cross-sectional associations over developmental stages, we also found the longitudinal relationship between blood zinc concentrations at preschool age and sleep quality in adolescence. The causal relationship in humans thus far is inconclusive due to limited evidence. In case of the predictive association we found, however, it seems reasonable to assume that lower zinc concentrations cause an increase in sleep-wake dysfunction in humans. With respect to indicators of sleep quality, early zinc status was predictive of later sleep efficiency. This finding seems inconsistent with the cross-sectional association with indicators of sleep duration and sleep disturbances in adolescence. However, sleep efficiency is the ratio of sleep duration to total time in bed [23], and to some extent reflects the level of sleep duration and sleep disturbances. Therefore, the longitudinal results support our cross-sectional findings in adolescence.

Although the effect size decreased in the longitudinal relationship with overall sleep quality relative to the cross-sectional association at adolescence, our findings suggest a possible long-lasting effect of suboptimal zinc status in early childhood on later sleep regulation. However, this might be a reflection of unobserved confounding in our study population due to drawbacks of the non-experimental design. For example, one study on iron deficiency anemia infants reported long lasting impact on sleep architecture in the follow-ups during childhood [35], which possibly resulted from the irreversible damage of iron deficiency to brain iron levels and sleep-related neurotransmitter systems [36,37,38]. Since interactions between iron and zinc occur in metabolic absorption [39], it is possible that our longitudinal findings are driven by unmeasured confounder of blood iron status. Further experimental research is warranted to examine whether the long-term biological action of zinc status is present in intrinsic sleep processes and sleep quality.

The strengths of this paper include two-wave longitudinal data from a preschool cohort, the large sample size, the use of blood zinc status and multiple validated parameters of sleep quality. Several potential limitations, however, should be taken into account when interpreting our results. First, sleep quality was based on parental-report and self-report. Although both CBCL and PSQI have good psychometric properties, future studies with objective sleep measures, such as polysomnography and actigraphy, are warranted to confirm the findings in this study. Another limitation is related to the challenge of applicability of our findings to other countries because sleep patterns and eating behaviors are socially and culturally constructed [40,41]. However, adolescents in Asian countries such as China need more attention due to higher rates of sleep deprivation, sleep impairment and daytime sleepiness than adolescents from other regions [11]. Similarly, high prevalence of micronutrient deficiency has been reported in Chinese children [16,42,43]. Chinese schoolchildren represent an opportunity for studying the relationship between zinc status and sleep quality. Additionally, the data of habitual dietary intake is not available in the present study. Therefore, we did not adjust for dietary nutrients, such as vitamin D, dodecanoic acid and total carbohydrate, which may relate to sleep outcomes [24,44]. Future studies should incorporate possible confounders of dietary intake and dietary habits into analyses. Finally, previous studies suggest possible interactions between zinc and copper [26], as well as zinc and iron [1], on sleep regulation. Consideration on future research should include the focus on the interactions between micronutrients and/or macronutrients that might be influential in the optimization of sleep quality [24].

## 5. Conclusions

Zinc is known to have important health implications in the development of brain function, neurotransmitter generation, metabolic reactions, physical maturation, as well as emotional and behavioral outcomes [46,47]. Despite the increasing interest in nutrient status and sleep impairment, few studies have specifically examined blood zinc concentrations in relation to sleep quality throughout childhood. Our findings from this population based sample suggest potential role of zinc status in optimizing sleep quality. Concurrent associations of blood zinc concentrations with sleep quality are present in adolescence but not at preschool age, indicating the moderating effect of development on the correlation. Blood zinc concentrations at preschool age are predictive of later sleep quality in adolescence. The potential links between zinc and sleep has significant implications for public health. It highlights the importance of detection and management of suboptimal zinc status as a target for the multifaceted and interrelated health issues of nutrition and sleep. Results from this study also warrant future research to examine the short term and long term effect of zinc status on sleep quality using randomized clinical trials, and to test the moderating effect of sleep quality between zinc and disease.

## References

[1] Kordas K., Siegel E.H., Olney D.K., Katz J., Tielsch J.M., Kariger P.K., Khalfan S.S., LeClerq S.C., Khatry S.K., Stoltzfus R.J. (2009). The effects of iron and/or zinc supplementation on maternal reports of sleep in infants from Nepal and Zanzibar. J. Dev. Behav. Pediatr..

[2] Rondanelli M., Opizzi A., Monteferrario F., Antoniello N., Manni R., Klersy C. (2011). The Effect of Melatonin, Magnesium, and Zinc on Primary Insomnia in Long-Term Care Facility Residents in Italy: A Double-Blind, Placebo-Controlled Clinical Trial. J. Am. Geriatr. Soc..

[3] Afaghi A., O’Connor H., Chow C.M. (2008). Acute effects of the very low carbohydrate diet on sleep indices. Nutr. Neurosci..

[4] Peuhkuri K., Sihvola N., Korpela R. (2012). Diet promotes sleep duration and quality. Nutr. Res..

[5] Ursin R. (2002). Serotonin and sleep. Sleep Med. Rev..

[6] Kordas K., Siegel E.H., Olney D.K., Katz J., Tielsch J.M., Chwaya H.M., Kariger P.K., LeClerq S.C., Khatry S.K., Stoltzfus R.J. (2008). Maternal reports of sleep in 6–18 month-old infants from Nepal and Zanzibar: Association with iron deficiency anemia and stunting. Early Hum. Dev..

[7] Peirano P., Algarín C., Garrido M., Algarín D., Lozoff B. (2007). Iron-deficiency anemia is associated with altered characteristics of sleep spindles in NREM sleep in infancy. Neurochem. Res..

[8] Grandner M.A., Jackson N., Gerstner J.R., Knutson K.L. (2013). Dietary nutrients associated with short and long sleep duration. Data from a nationally representative sample. Appetite.

[9] Chung K.F., Cheung M.M. (2008). Sleep-wake patterns and sleep disturbance among Hong Kong Chinese adolescents. Sleep.

[10] Eaton D.K., McKnight-Eily L.R., Lowry R., Perry G.S., Presley-Cantrell L., Croft J.B. (2010). Prevalence of insufficient, borderline, and optimal hours of sleep among high school students–United States, 2007. J. Adolesc. Health.

[11] Gradisar M., Gardner G., Dohnt H. (2011). Recent worldwide sleep patterns and problems during adolescence: A review and meta-analysis of age, region, and sleep. Sleep Med..

[12] National Sleep Fundation 2006 Sleep in America Poll. http://sleepfoundation.org/sites/default/files/2006_summary_of_findings.pdf.

[13] Bryan J., Osendarp S., Hughes D., Calvaresi E., Baghurst K., van Klinken J.W. (2004). Nutrients for cognitive development in school-aged children. Nutr. Rev..

[14] Liu J., McCauley L.A., Zhao Y., Zhang H., Pinto-Martin J. (2010). Cohort profile: The China Jintan child cohort study. Int. J. Epidemiol..

[15] Liu J., McCauley L., Leung P., Wang B., Needleman H., Pinto-Martin J., Group J.C. (2011). Community-based participatory research (CBPR) approach to study children’s health in China: Experiences and reflections. Int. J. Nurs. Stud..

[16] Liu J., Ai Y.X., Hanlon A., Shi Z., Dickerman B., Compher C. (2011). Micronutrients deficiency and associated sociodemographic factors in Chinese children. World J. Pediatr..

[17] Dong S., Zhu Z., Liu W. (2001). Determination of Ca, Mg, Fe, Mn, Cu, and Zn in lixin pill by atomic absorption spectrophotometry. Guang Pu Xue Yu Guang Pu Fen Xi.

[18] Zhao T., Chen T., Qiu Y., Zou X., Li X., Su M., Yan C., Zhao A., Jia W. (2009). Trace element profiling using inductively coupled plasma mass spectrometry and its application in an osteoarthritis study. Anal. Chem..

[19] Liu J., Zhou G., Wang Y., Ai Y., Pinto-Martin J., Liu X. (2012). Sleep problems, fatigue, and cognitive performance in Chinese kindergarten children. J. Pediatr..

[20] Achenbach T., Rescorla L. (2001). Manual for the ASEBA school-age forms & profiles: An integrated system of multi-informant assessment. Research Center for Children, Youth, & Families.

[21] Liu X., Sun Z., Uchiyama M., Shibui K., Kim K., Okawa M. (2000). Prevalence and correlates of sleep problems in Chinese schoolchildren. Sleep.

[22] Zhou H.Q., Shi W.B., Wang X.F., Yao M., Cheng G.Y., Chen P.Y., Li D.G. (2012). An epidemiological study of sleep quality in adolescents in South China: A school-based study. Child.

[23] Buysse D.J., Reynolds C.F., Monk T.H., Berman S.R., Kupfer D.J. (1989). The Pittsburgh Sleep Quality Index: A new instrument for psychiatric practice and research. Psychiatry Res..

[24] Sato-Mito N., Sasaki S., Murakami K., Okubo H., Takahashi Y., Shibata S., Yamada K., Sato K. (2011). The midpoint of sleep is associated with dietary intake and dietary behavior among young Japanese women. Sleep Med..

[25] Liu J., Hanlon A., Ma C., Zhao S.R., Cao S., Compher C. (2014). Low Blood Zinc, Iron, and Other Sociodemographic Factors Associated with Behavior Problems in Preschoolers. Nutrients.

[26] Song C.H., Kim Y.-H., Jung K.I. (2012). Associations of Zinc and Copper Levels in Serum and Hair with Sleep Duration in Adult Women. Biol. Trace Element Res..

[27] Bhatnagar S., Taneja S. (2001). Zinc and cognitive development. Br. J. Nutr..

[28] Takeda A., Minami A., Seki Y., Oku N. (2004). Differential effects of zinc on glutamatergic and GABAergic neurotransmitter systems in the hippocampus. J. Neurosci. Res..

[29] Turgeon S.M., Albin R.L. (1992). Zinc modulates GABA B binding in rat brain. Brain Res..

[30] Golub M.S., Takeuchi P.T., Keen C.L., Hendrickx A.G., Gershwin M.E. (1996). Activity and attention in zinc-deprived adolescent monkeys. Am. J. Clin. Nutr..

[31] Carney P.R., Berry R.B., Geyer J.D. (2005). Clinical Sleep Disorders.

[32] Kordas K., Casavantes K.M., Mendoza C., Lopez P., Ronquillo D., Rosado J.L., Vargas G.G., Stoltzfus R.J. (2007). The association between lead and micronutrient status, and children’s sleep, classroom behavior, and activity. Arch. Environ. Occup. Health.

[33] Iglesia I., Doets E.L., Bel-Serrat S., Roman B., Hermoso M., Peña Quintana L., Garcia-Luzardo M.D.R., Santana-Salguero B., Garcia-Santos Y., Vucic V. (2010). Physiological and public health basis for assessing micronutrient requirements in children and adolescents. The EURRECA network. Mater. Child Nutr..

[34] Ohayon M.M., Carskadon M.A., Guilleminault C., Vitiello M.V. (2004). Meta-analysis of quantitative sleep parameters from childhood to old age in healthy individuals: Developing normative sleep values across the human lifespan. SLEEP.

[35] Peirano P.D., Algarín C.R., Garrido M.I., Lozoff B. (2007). Iron deficiency anemia in infancy is associated with altered temporal organization of sleep states in childhood. Pediatr. Res..

[36] Beard J., Erikson K.M., Jones B.C. (2003). Neonatal iron deficiency results in irreversible changes in dopamine function in rats. J. Nutr..

[37] Rao R., Tkac I., Townsend E.L., Gruetter R., Georgieff M.K. (2003). Perinatal iron deficiency alters the neurochemical profile of the developing rat hippocampus. J. Nutr..

[38] Felt B.T., Beard J.L., Schallert T., Shao J., Aldridge J.W., Connor J.R., Georgieff M.K., Lozoff B. (2006). Persistent neurochemical and behavioral abnormalities in adulthood despite early iron supplementation for perinatal iron deficiency anemia in rats. Behav. Brain Res..

[39] Singh M. (2004). Role of micronutrients for physical growth and mental development. Indian J. Pediatr..

[40] Jenni O.G., O’Connor B.B. (2005). Children’s sleep: An interplay between culture and biology. Pediatrics.

[41] Eertmans A., Baeyens F., van den Bergh O. (2001). Food likes and their relative importance in human eating behavior: Review and preliminary suggestions for health promotion. Health Educ. Res..

[42] Zhao T.T., Chen B., Wang H.P., Wang R., Zhang H. (2013). Evaluation of toxic and essential elements in whole blood from 0- to 6-year-old children from Jinan, China. Clin. Biochem..

[43] Qin Y., Melse-Boonstra A., Shi Z., Pan X., Yuan B., Dai Y., Zhao J., Zimmermann M.B., Kok F.J., Zhou M. (2009). Dietary intake of zinc in the population of Jiangsu Province, China. Asia Pacific J. Clin. Nutr..

[44] Grandner M.A., Jackson N., Gerstner J.R., Knutson K.L. (2014). Sleep symptoms associated with intake of specific dietary nutrients. J. Sleep Res..

[45] Katz D.L. (2012). Nutrition in Clinical Practice: A Comprehensive, Evidence-Based Manual for the Practitioner.

[46] Liu J., Raine A. (2006). The effect of childhood malnutrition on externalizing behavior. Curr. Opin. Pediatr..

